# *Nomen est omen*: why we need to rename ‘antimicrobial resistance’

**DOI:** 10.1093/jacamr/dlaa067

**Published:** 2020-08-11

**Authors:** Eva M Krockow

**Affiliations:** Department of Neuroscience, Psychology and Behaviour, University of Leicester, Leicester LE1 7RH, UK

## Abstract

The naming of diseases is a critical aspect of public health communication. In light of the recent renaming of the ‘Wuhan novel coronavirus’ to COVID-19, the names of other health threats must be reviewed. In particular, a new name is urgently needed for the global challenge typically referred to as ‘antimicrobial resistance’. The current name is inconsistently used, difficult to pronounce and lacks meaning for lay audiences. It also fails to express the magnitude of the phenomenon’s potential consequences for human medicine. This article reviews and evaluates key findings from several cross-disciplinary streams of research on the psycholinguistic properties of names. These include early psychology literature pertaining to the concept of ‘word attensity’, recent cognitive research on ‘processing fluency’ in the context of word recognition, and relevant marketing literature examining the components of successful branding strategies. Three key criteria—pronounceability, meaningfulness and specificity—are found to influence the perception of names and these are discussed in the context of antimicrobial resistance. The article demonstrates that the current term of ‘antimicrobial resistance’ falls short with regard to all three criteria and concludes with specific recommendations for the creation of a new name. Only the strategic choice of a single term that is (i) short and easy to pronounce; (ii) intuitively meaningful to lay audiences and indicative of the existential threat linked to antimicrobial resistance; and (iii) uniquely associated with the topic of antimicrobial resistance is likely to bring about overdue change in the global discussion of antimicrobial resistance.

In February 2020, the WHO announced an official name for a rapidly spreading viral disease. The disease, which had previously been referred to as ‘nCoV-2019’ or simply ‘Wuhan novel coronavirus’ was renamed to ‘COVID-19’.^1^ This was a decisive move to provide a more accurate label for a disease caused by a specific virus strain. It also took action against some of the sociopolitical consequences of the disease, including stigmatization of the geographical location associated with its outbreak.^1^ The renaming of illnesses is not a novel occurrence;^2^ the disease formerly known as ‘gay-related immune deficiency’ (GRID) and sometimes offhandedly referred to as ‘gay cancer’ underwent a name change in the 1980s to reflect improved understanding about the risk population and avoid stigmatization of homosexual men. The new name—HIV/AIDS—has now been used consistently for many decades.^3^ Similarly, many diseases initially named after scientists or places of discovery were subsequently renamed to avoid negative connotations for the individuals or places in question.^2^ Finally, some changes in terminology have been driven by strategic shifts in communication. A recent example is that of ‘climate change’, with some politicians and media outlets adopting more persuasive language including ‘climate emergency’ and ‘global heating’ to reflect the urgency and magnitude of its threat.^4^

Identifying suitable names for public health threats is a key prerequisite for successful science communication with lay audiences. The significance of names has long been recognized in popular culture. Bemoaning the tragic consequences of her lover’s family name, Shakespeare’s Juliet famously exclaimed: ‘What’s in a name?’ Similarly, the Latin proverb *nomen est omen*—originally attributed to Roman playwright Plautus—proclaims a causal link between a person’s name and their destiny.

Despite the overwhelming evidence on the importance of names, the scientific community continues to fall short in the coining of successful terminology to disseminate scientific discoveries about diseases or raise awareness about global challenges. One such challenge is antimicrobial resistance (commonly abbreviated as AMR).

## Antimicrobial resistance

By 2050, more people will die from antimicrobial resistance (AMR) than currently die from cancer.^5^ AMR refers to mutations in microbes, such as bacteria, viruses and fungi, that render them unresponsive to existing medical treatment. Focusing on bacteria, an important subcategory of AMR is antibiotic resistance. While AMR is a natural process, it is greatly exacerbated by the persistent overuse of antimicrobial medicines such as antibiotics. As a result, common infections (e.g. urinary tract infections, pneumonia) become more difficult or even impossible to treat.^6^ If left unaddressed, drug-resistant infections are estimated to incur global costs of 100 trillion USD over the next 35 years.^5^ As a result, AMR has been described as one of the gravest challenges of 21st century medicine,^7^ and a complete loss of antimicrobial drug efficacy has been likened to a return to the medical dark ages.^8^ Indeed, the threat to humanity imposed by AMR may be on a par with that of climate change.^9^

Given the undebatable importance of the problem, AMR continues to receive astonishingly little media coverage.^10^ Consequently, public and even physicians’ knowledge about AMR remains low.^11^^,^^12^ Politically salient topics of the past few years included Brexit and Europe, immigration, benefit fraud and climate change,^13^ while rising concerns about the medical impact of AMR continue to be sidelined. For example, a comprehensive media search of six highly circulated UK newspapers found that up until 30 June 2018 only 201 articles had been published that focused on the problem of AMR.^14^ By comparison, the related health threat of sepsis was covered by more than twice the number of articles in the same time frame.^14^ Several reasons have been suggested for the comparative lack of media attention, and this includes the abstract, scientific nature of AMR, which complicates communication to lay audiences.^10^

Another challenge for successful AMR communication is its name and all related terminology. This includes ‘antimicrobial stewardship’—a term broadly referring to measures aimed at reducing the overuse of antimicrobial agents, which is difficult to understand for prescribers, let alone the general public. Also problematic is the common yet ambiguous term ‘antibiotic’ that is often used to mean ‘antibacterial agent’ and might be contributing to the public confusion about the role of ‘antibiotics’ for treating bacterial versus viral infections. To help communicate a large range of subtly different, yet interrelated, concepts, there appears to be a need for a whole new public language related to the global health challenge of AMR.

Given the scope of this short review, however, the remainder of the article will focus on specifically evaluating the central term ‘antimicrobial resistance’. In this context, the UK Wellcome Trust Initiative ‘Re-branding Resistance’ has made important contributions by outlining the problematic inconsistency of the name when disseminating information about AMR.^15^ Media terms for the health threat vary greatly and, for the English language context, the six most common names were identified as ‘superbugs’, ‘antibiotic resistance’, ‘antimicrobial resistance’, ‘drug-resistant infections’, ‘antibiotic-resistant bacteria’ and ‘AMR’.^15^ However, more name variations are likely to exist and a meaningful comparison of the different terms will only be possible once a comprehensive list has been established. Overall, the variability in language reduces translatability of the terminology into different languages and complicates consistent storytelling.

Consistency undoubtedly plays a crucial role in successful communication. However, other aspects such as the psycholinguistic properties of a name are just as likely to influence public awareness and understanding. The importance of such properties has been demonstrated by research from the scientific fields of linguistics, marketing and psychology, which attests to the existence of measurable features shaping the subjective experience of different names.

## Attensity

Stemming from a body of fundamental psychology research from the 1960s, the term ‘attensity’ was introduced to describe a word’s potential to engage people, capture their attention and improve subsequent memory of the word.^16^^,^^17^ Attensity was first explored in the context of cognitive experiments involving variations of the Stroop task,^17^ which tested participants’ abilities to name the ink colour of words that spelled out mismatching colours. The distracting influence of written words was found to be larger for some words than others, and researchers attributed this to different dimensions of a word’s attensity. These included a word’s pronounceability, frequency of its phonetic components, length, meaningfulness, concreteness and specificity.

Pronounceability refers to the ease of verbalizing a word, with research showing that words of higher pronounceability are processed more quickly,^18^ typically appear to be more familiar^19^ and thus seem more important. This in turn improves recall for these words and may even affect gut feelings about the terms’ truthfulness.^20^ Similarly, the frequency of a word’s components may affect people’s perception of a word. In a word recognition task, for example, it was found that words with phoneme clusters more frequent in the English vocabulary were recalled more easily.^21^ Another aspect related to pronounceability is word length, with longer words often being harder to articulate and more difficult to remember.^22^

Meaningfulness and concreteness describe a word’s power to associate meaning. This may include the ease with which related mental images are retrieved or the availability of meaningful context to make sense of the word.^23^ Concreteness has been found to ease recall^24^ and increase sensory focus to the word.^16^ A similar concept is that of specificity, which refers to a word’s unique meaning.^25^ Specificity is decreased if a word can be associated with multiple meanings and therefore becomes ambiguous in its descriptive power.

## Processing fluency

An updated and more comprehensive perspective on word attensity was provided by recent research on so-called processing fluency. The term refers to a subjective feeling of ease experienced when processing a stimulus such as a written word, and is often related to shorter reading or response times.^26^ Factors proposed to influence processing fluency are similar to those previously studied in the context of attensity. More readable (i.e. short and simple) stimuli^27^ that are easy to pronounce,^20^^,^^28^ frequently repeated^29^ and presented in legible fonts^30^ generally lead to high levels of processing fluency. Experimental studies assessed self-reported subjective ratings of processing fluency^31^ and found that the concept was able to predict feelings of familiarity,^30^ risk,^28^^,^^32^ likability^33^^,^^34^ and illusory truth^29^ with regard to the processed stimuli.

The concept of processing fluency is frequently applied to design materials for less proficient readers (e.g. making recommendations on letter spacing or font types^35^). It has also received attention in the context of ‘fake news’, with scholars arguing that misinformation can be spread more easily through the strategic manipulation of processing fluency associated with its contents.^36^

## Branding

In addition to the psychological research on the cognitive processes involved in word perception, literature from the fields of branding and marketing have highlighted the importance of word features such as memorability and uniqueness.^37^ Memorability is typically determined by the existence of ‘semantic imbeds’,^38^ i.e. meaningful word components that are recognizable and may convey inherent messages. An example of this includes the brand name ‘Facebook’, which refers to a widely used social media site. The name was created by joining two existing words with semantic meaning, thus achieving high memorability.^37^

Uniqueness, on the other hand, refers to infrequent usage of the term in question^37^ and the existence of no alternative word meanings. A recent example of unique terminology may be the term ‘Brexit’, which was specifically created to describe the political process of the UK leaving the EU. The term rose to significance during the time of the EU referendum in 2016 and even earned the Collins Dictionary title ‘word of the year’.^39^

The combined literature from different fields of research demonstrates the importance of names and terminology on people’s perceptions of a particular topic. Psycholinguistic word properties had varying scientific terms across different fields, but they appear to fall into three thematic categories: (i) pronounceability and readability, referring to the name’s lexical attributes (e.g. letter composition, vowel/consonant ratio, word length, phonetical frequency); (ii) meaningfulness, concreteness and memorability, referring to a name’s power of evoking intuitive context and meaning; and (iii) specificity and uniqueness, referring to a word’s unambiguous meaning and novel linguistic aspects.

## How pronounceable, meaningful and specific is ‘antimicrobial resistance’?

Unfortunately, the name ‘antimicrobial resistance’ appears to fall short when measured against all these criteria and this might explain why the term fails to capture and sustain the international attention it beckons. The full term ‘antimicrobial resistance’ is characterized by a high word length of nine syllables, which is difficult to pronounce and contains infrequent word components. Due to its abstract, scientific nature, the term also lacks intuitive meaningfulness for lay populations and may therefore be difficult to remember. While ‘antimicrobial resistance’ can be considered less stigmatizing than previous disease names such as ‘Wuhan novel coronavirus’ or ‘GRID’, the current terminology fails to signal the existential threat associated with the phenomenon and may lead lay audiences to underestimate its risks. Finally, given the inaccurate use of the umbrella term ‘antimicrobial resistance’ to describe the more specific phenomena of ‘antibiotic resistance’ or ‘antifungal resistance’, the term also has limited specificity. The frequently used acronym ‘AMR’ may be even more problematic; the three letter abbreviation is highly ambiguous because it can also refer to ‘anisotropic magnetoresistance’, ‘automatic meter reading’ or an Arabic male name, to list but a few.^40^ Indeed, the previously cited study of AMR media coverage found that more than half of the initial search results on ‘AMR’ referred to topics other than antimicrobial resistance.^14^

Following on from the above analysis, it appears that the communication of AMR is challenged by the psycholinguistic properties of its name. Drawing on previous research findings, the consequences might be far-reaching, for example influencing the attention paid to the health threat and even the feelings of truth or risk associated with relevant media communications.

While previous scholars have called for a rebranding of AMR, for example suggesting to build consensus around the consistent use of one existing term (e.g. drug resistance),^15^ this suggestion is unlikely to satisfy the need for a powerful new name fulfilling the key criteria outlined above. In the context of generating the disease name ‘COVID-19’, WHO chief Tedros Adhanom Ghebreyesus stated: ‘We had to find a name that did not refer to a geographical location, an animal, an individual or group of people, and which is also pronounceable and related to the disease.’^1^ Similar multidimensional considerations are necessary when identifying a new term for AMR. The strategically created disease name ‘COVID-19’ is an amalgamation of the words ‘corona’, ‘virus’ and ‘disease’, with the number specifying the year of its outbreak. A similar approach may be required to replace the problematic term AMR.

Based on the review above, I propose three recommendations for the overdue renaming process of AMR: (i) reduce variability of the names of AMR to one term that is universally used and understood; (ii) choose a term that is (a) short and easy to pronounce, (b) intuitively meaningful to lay audiences and indicative of the existential threat linked to AMR and (c) uniquely associated with the topic of AMR; and (iii) ensure that the name translates unambiguously across different languages and cultures.

The rebranding of a global health threat like AMR requires a large-scale, unified campaign led by an interdisciplinary team of psychologists and clinicians and spearheaded by an international organization such as the WHO. In addition to the specific AMR name changes suggested above, a wider evaluation and revision of all related terminology such as ‘antimicrobial stewardship’ is likely to be necessary. The success of such language change will undoubtedly determine the international response to AMR over many years to come.

## Funding 

This work was supported by a Leicester-Wellcome Trust ISSF Fellowship (reference 204801/Z/16/Z).

## Transparency declarations

None to declare.
